# Fosfomycin Susceptibility Testing Using Commercial Agar Dilution Test

**DOI:** 10.1128/spectrum.02504-21

**Published:** 2022-03-30

**Authors:** Peter D. Croughs, Michelle Konijnendijk-de Regt, Erlangga Yusuf

**Affiliations:** a Department of Medical Microbiology and Infectious Diseases, Erasmus Medical Center, Rotterdam, the Netherlands; University of Utah and ARUP Laboratories

**Keywords:** fosfomycin, antimicrobial susceptibility testing, agar dilution

## Abstract

The reference standard for fosfomycin antimicrobial susceptibility testing (AST) is agar dilution, but it is laborious and is not routinely used in diagnostic microbiology. In this study, we evaluated the performance of a ready-to-use commercially available agar dilution kit for fosfomycin AST (Liofilchem Diagnostics). We compared this kit with the reference standard agar dilution, performed according to the Clinical & Laboratory Standards Institute (CLSI) in 229 clinical isolates. The isolates were selected to represent both Gram-positive and Gram-negative microorganisms, with various MIC values. It consisted of 43 enterococci (E. faecalis *n* = 16, E. faecium *n* = 27), 13 methicillin-resistant S. aureus (MRSA), 118 Enterobacterales (Escherichia coli *n* = 94, Klebsiella pneumoniae *n* = 20, and Enterobacter cloacae complex *n* = 4), 55 Pseudomonas aeruginosa, and three ATCC isolates. Using CLSI breakpoints for enterococci for oral treatment of urinary tract infections, European Committee on Antimicrobial Susceptibility Testing (EUCAST) breakpoints for intravenous dosing for Enterobacterales and Staphylococci, and epidemiological cutoff value for P. aeruginosa, the essential agreement was 87.5%, and 99.6% after discrepancy resolution. There was no very major error, and 1.9% major error before, and 0.9% major error after resolution of discrepancies. The commercial test showed 100% reproducibility. In conclusion, in comparison to the reference standard, the ready-to-use commercially available agar dilution kit for fosfomycin AST showed excellent performance.

**IMPORTANCE** Fosfomycin is increasingly needed to treat infection due to multidrug resistant microorganisms. Yet, the antimicrobial susceptibility test (AST) of fosfomycin is fraught with difficulties or often laborious to perform. An easy-to-use AST method for fosfomycin is thus needed. In this study, we showed that the ready-to-use commercially available agar dilution kit, in comparison to the reference standard, showed excellent performance.

## INTRODUCTION

Fosfomycin is a broad-spectrum antibiotic produced by Streptomyces fradiae discovered in Spain in 1969 (1). It is a bactericidal antibiotic and exerts its effect by inactivation of the enzyme UDP-*N*-acetylglucosamine enolpyruvyl transferase (MurA) which is involved in peptidoglycan synthesis, that consequently leads to the inhibition of bacterial cell wall synthesis. The main form of administration of fosfomycin is orally, to treat uncomplicated urinary tract infections. Yet, its parenteral use has gained interest due to the increased number of infections caused by multidrug-resistant microorganisms (2). There is also an increased interest in the use of fosfomycin as an anti-biofilm agent in prosthetic joint infection caused by Staphylococcus aureus (3). Resistance against fosfomycin can occur even during treatment, especially in case of P. aeruginosa infections (4). Antimicrobial susceptibility testing (AST) for fosfomycin is thus important, but it is fraught with difficulty. The reference standard for fosfomycin AST is agar dilution with addition of 25 mg/L glucose-6-phosphate according to both Clinical and Laboratory Standards Institute (CLSI) (5) and European Committee on Antimicrobial Susceptibility Testing (EUCAST) (6). The addition of glucose-6-phosphate is to compensate the reduction of fosfomycin uptake by bacteria due to the presence of glucose and phosphate in Mueller-Hinton agar (7). However, agar dilution is not routinely used in diagnostic microbiology labs since it is labor-intensive. Other methods have been proposed and used such as disk using 200 μg disk containing 50 μg glucose-6-phosphate, gradient diffusion test, Vitek2, and Phoenix, but none of them performed satisfactorily due to poor detection of resistant isolates and very high error rates (8). For examples, the error rates was as high as 23.3% for Etest, and 12.9% for disk diffusion in Escherichia coli.

Recently, a ready-to-use commercially available agar dilution kit for fosfomycin AST (Liofilchem Diagnostics) has been brought to the market. The plate consists of 12 wells, containing fosfomycin incorporated into agar medium in 11 2-fold dilutions (0.25 to 256 mg/L), and a well with growth control. The aim of the present study is to evaluate the performance of this AST in comparison to the reference standard, making use of CLSI breakpoints for enterococci for oral treatment of urinary tract infections, European Committee on Antimicrobial Susceptibility Testing (EUCAST) breakpoints for intravenous dosing for Enterobacterales and Staphylococci, and epidemiological cutoff value for Pseudomonas aeruginosa.

## RESULTS

### Distribution of commercial fosfomycin agar dilution test.

**FIG 1:**
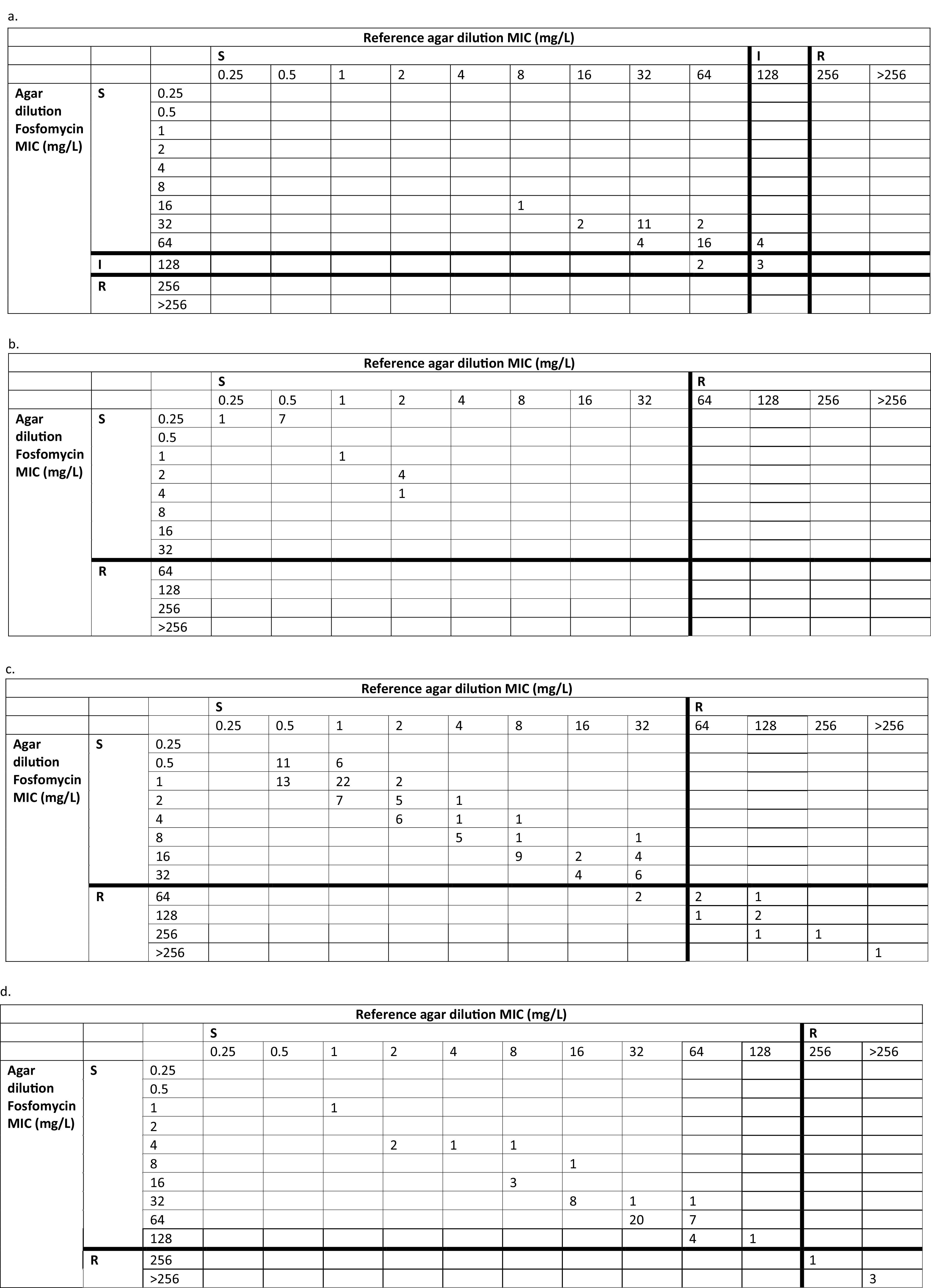
Scatter diagram of fosfomycin susceptibility testing using commercial agar dilution test in comparison to reference standard using applicable breakpoints. (a) *Enterococcus* species isolates, (b) Methicllin resistant Staphylococcus aureus, (c) Enterobacterales, (d) Pseudomonas aeruginosa.

The MIC distribution plots of included isolates as determined by the commercial test are presented in Fig. 1 (a to d). According to the used breakpoints, all enterococcus isolates were susceptible, except seven (15.6%) which were intermediate according to the reference agar dilution. Of these seven isolates, three also tested intermediate by the commercial test. Two isolates tested intermediate by the commercial test, while susceptible by reference test. All MRSA isolates (*n* = 14) were susceptible according to reference and commercial test. and For of Enterobacterales, nine (8%) were resistant by both methods, and two isolates tested resistant by commercial method, while susceptible by reference method. All P. aeruginosa isolates were susceptible by both methods, except and four (7%), which were resistant by both methods. of the P. aeruginosa isolates were deemed as resistant.

### Performance of commercial agar dilution test.

In 98.7% (229 of 232 isolates) the result of the commercial fosfomycin test differed within ±2 dilutions, from reference method. Of these isolates, 203 (87.5%) had a MIC within ±1 dilution difference compared to the reference standard (essential agreement). When the fosfomycin AST commercial test of 29 isolates with > ±1 dilution difference was repeated from the same inoculum together with the reference standard, the EA was 99.6% (231 of 232 isolates). Only one isolate, a K. pneumoniae with the MIC of 8 mg/L had a difference in MIC of 2 dilutions (MIC according to reference test was 32 mg/L). The CA of the commercial test compared with reference standard was 95.6% (222 of 232 isolates) before and 95.7% (224 of 232 isolates) after resolution of discrepancies (Table 1).

**TABLE 1:** Essential, categorical agreement, and errors of the commercial agar Dilution test in comparison to the reference test using breakpoints described in Table 3

	Doubling dilution(s) difference n/n (%)		Categorical agreement n/n (%)	Major error		Minor error n/n (%)^*a*^
Microorganism	Within one doubling dilution (essential agreement)	Within two doubling dilution		Before resolving discrepancies n/n (%)	After resolving discrepancies n/n (%)	
*Enterococcus* spp.	44/45 (97.8)	45/45 (100)	39/45 (86.7)	0/38 (0)	0/38 (0)	6/45 (13.3)
Staphylococcus *spp.*	14/14 (100)	14/14 (100)	14/14 (100)	0/14 (0)	0/14 (0)	n.a.
Enterobacterales	99/118 (83.9)	115/118 (97.5)	114/118 (96.6)	4/110 (3.6)	2/109 (1.8)	n.a.
Pseudomonas aeruginosa	46/55 (83.6)	55/55 (100)	55/55 (100)	0/52 (0)	0/51 (0)	n.a.
Total	203/232 (87.5)	229/232 (98.7)	222/232 (95.7)	4/214 (1.9)	2/212 (0.9)	6/45 (13.3)

a n.a., not applicable.

There was no VME, and proportion of ME was 1.9% (4 of 214 isolates, all E. coli) and 0.9% (2 of 212 isolates, both E. coli) before and after resolution of discrepancies, respectively. This was the only category in which the proportion of errors changed due to the resolution of discrepancies. In 6 of 232 isolates (all enterococci), an intermediate MIC was shown by either one test, and susceptible or resistant by the other test, giving mE proportion of 13.3% (6 out of 45 Enterococci isolates).

Commercial test showed 100% reproducibility. The testing of E. faecalis ATCC 29212, S. aureus ATCC 29213, E. coli ATCC 25922, and P. aeruginosa ATCC 27853, on five consecutive days resulted in MICs not differing more than one doubling dilution (Table 2), and 6-fold testing of P. aeruginosa ATCC 27853 on the same day by the commercial test resulted in exactly the same MIC (4 mg/L).

**TABLE 2:** MICs of quality control isolates on five consecutive days

Isolate	MIC (mg/L)				
Enterococcus faecalis ATCC 29212	32	32	32	32	32
Escherichia coli ATCC 25922	1	1	1	2	1
Pseudomonas aeruginosa ATCC 27853	4	4	8	4	4
Staphylococcus aureus ATCC 29213	1	2	2	2	2

## DISCUSSION

The need for valid AST for fosfomycin will further rise due to increased interest in this antibiotic to treat infections caused by multidrug resistant Gram-negative organisms (9, 10). The interest in the use of fosfomycin has also increased due to its possible anti-biofilm effect where there may be a role for fosfomycin in combination with another antibiotic (3).

In this study, we showed the excellent performance according to CLSI requirements (11) (EA ≥90%, CA ≥90%, VME ≤1.5%, ME ≤3.0%) of the first commercially available agar dilution for fosfomycin. This EA was obtained, after resolution of discrepancies, by repeating the test from the same inoculum. This performance was calculated using EUCAST breakpoints for staphylococci and Enterobacterales, CLSI breakpoints (UTI) for enterococci, and epidemiological cutoff value in case of P. aeruginosa.

Alternatives for fosfomycin AST have often shown to have limited performance. In 57 extended spectrum beta-lactamase (ESBL) producing isolates, Etest has shown to have an EA of 57%, where Etest mostly underestimated the MIC (12). Disk diffusion showed CA as low as 66% in 68 K. pneumoniae isolates (using EUCAST breakpoint) (13). In the same study, broth microdilution showed CA as low as 67% in 64 E. coli isolates. Another difficulty is regarding the interpretation of commercial disk and Etest due to colonies within zones that differs between EUCAST and CLSI. In EUCAST reading guidelines, the colonies within zones should be disregarded, while those of CLSI do not disregard these colonies.

Next to the performance, the commercial agar dilution is also convenient, as it is ready to use, in contrary to the labor-intensive preparation of the in-house agar dilution method. Therefore, this method might be considered to be implemented in the routine practice. However, since the commercial panel is costly, and has a limited expiration time of 4 months, a routine laboratory needs to plan whether it should invest in having this commercial test or to send the AST to a larger lab. The latter comes at the cost of delaying appropriate treatment for the patient. An alternative offered by the company is to buy a plate with a custom-made dilution range. Based on our study an option would be a plate designed for three isolates, with 3 times a dilution range, from 32 mg/L to 128 mg/L, and 3 controls.

Our results show similarities with previous studies on Liofilchem agar dilution assay. The agreement is excellent, but there is a trend in overcalling resistance in the Enterobacterales when using this commercial product (14, 15).

The strength of this study was the use of a large number of samples, and the inclusion of a large number of enterococci unlike previous publications using Liofilchem agar dilution assay (14, 15). A limitation is the limited number of MRSA isolates and the use of the historical results of reference method. Only in case of discrepancy, both methods repeated from one inoculum.

In conclusion, we showed that the commercial agar dilution is a valid AST method in testing fosfomycin susceptibility.

## MATERIALS AND METHODS

### Bacterial isolates.

For this evaluation a collection of 232 isolates was used, of which 229 clinical isolates and three ATCC isolates (Enterococcus faecalis ATCC 29212, E. faecium ATCC 35667 and S. aureus ATCC 43300). Clinical isolates were derived from various anatomical sites, collected as part of standard care from patients admitted to Erasmus Medical Center University Hospital, Rotterdam, and Haaglanden Medical Center, The Hague, The Netherlands. The fosfomycin MIC of these clinical isolates had been determined at the moment of isolation between 2016 and 2021.

The clinical isolates were selected to represent Gram-positive and Gram-negative microorganisms with various MIC values and consisted of 43 enterococci (E. faecalis *n* = 16, E. faecium *n* = 27), and 13 methicillin-resistant S. aureus (MRSA), 118 Enterobacterales (E. coli *n* = 94, Klebsiella pneumoniae *n* = 20, and Enterobacter cloacae complex *n* = 4), and 55 Pseudomonas aeruginosa. None of the Enterobacterales and P. aeruginosa isolates harbored genes coding for carbapenem producing enzymes.

### Reference standard.

The reference standard used in this study was in-house agar dilution method as recommended by CLSI guidelines (16) in which cation-adjusted Mueller-Hinton agar (BD BBL, Sparks, MD, USA) supplemented with 25 mg/L of glucose-6-phosphate (Roche Diagnostics GmbH, Germany) was used. Fosfomycin (InfectoPharm, Germany) incorporated into agar was tested over a range of dilutions 0.25 to 256 mg/L. Overnight cultures, grown on blood agar (BD, Heidelberg, Germany), were used to prepare 0.5 McFarland suspensions, which were diluted 1:10. Subsequently a 2 μL spot was inoculated on a petri dish containing 20 mL agar. Per petri dish, 12 isolates were inoculated. Plates were read after overnight incubation in ambient air, at 35°C, and hereby pinpoint colonies and a thin film of growth were neglected.

### MIC determination of fosfomycin using commercial agar dilution panel.

The panel was used according to the manufacturer’s instructions. The inoculum was prepared in the same way as inoculum preparation for reference standard. Onto the agar, 2 μL of the suspension was inoculated with one spot per well, starting from the growth-control well to the highest concentration of fosfomycin. Plates were read after overnight incubation in ambient air, at 35°C according to manufacturer’s instruction, in which pinpoint colonies and a thin film of growth were neglected.

### Statistical analysis.

Accuracy, defined as the closeness of the result obtained with the commercial test to the reference standard (17) was determined by calculating essential agreement (EA) and categorical agreement (CA). EA was defined as MIC obtained with commercial test within ±1 doubling dilutions from the reference MIC. When the discrepancies were > ±1 doubling dilutions, both tests were repeated (once) from the same inoculum. CA was defined as total number of isolates tested using the commercial test that yielded the same categorical interpretation as the reference standard according to EUCAST Breakpoint Tables version 11.0 for intravenous dosing (resistant >32 mg/L for Enterobacterales and Staphylococcus spp.) (Table 3).

**TABLE 3:** Breakpoints used in this study

	Minimum inhibitory cconcetration (MIC), mg/L			
Microorganism	Susceptible (≤)	Intermediate	Resistent (>)	Breakpoints
*Enterococcus* spp.	64	128	128	CLSI for oral treatment of urinary tract infections
Staphylococcus *spp.*	32		32	EUCAST for intravenous dosing
Enterobacterales	32		32	EUCAST for intravenous dosing
Pseudomonas aeruginosa	128		128	Epidemiological cutoff value

Since no EUCAST breakpoints are available for P. aeruginosa and enterococci, P. aeruginosa was considered resistant if MIC >128 mg/L based on epidemiological cutoff value, and for enterococci CLSI breakpoints for urinary tract infections were used (susceptible ≤64 mg/L, intermediate susceptible 128 mg/L, resistant ≥256 mg/L).

Further, we calculated very major error (VME), major error (ME), and minor error (mE) proportions. VME was defined as resistant by reference test, but susceptible by commercial test (false susceptible). ME was defined as susceptible by reference test but resistant by commercial test. mE was defined as intermediate by one test, and susceptible or resistant by the other test. Due to the used breakpoints, mE was only calculated for enterococci.

Reproducibility was evaluated by testing of E. faecalis ATCC 29212, S. aureus ATCC 29213, E. coli ATCC 25922, and P. aeruginosa ATCC 27853, on five consecutive days, and the latter was also tested 6-fold on 1 day. The tests were deemed as reproducible when the MICs were within ± one doubling dilution.
